# Modeling the economic burden of postpartum hemorrhage due to substandard uterotonics in Ghana

**DOI:** 10.1371/journal.pgph.0003181

**Published:** 2024-06-20

**Authors:** Kiara Bautista, Yi-Fang (Ashley) Lee, Colleen R. Higgins, Petra Procter, Sara Rushwan, Abraham Baidoo, Kofi Issah, Chris Opoku Fofie, A. Metin Gülmezoglu, Lester Chinery, Sachiko Ozawa

**Affiliations:** 1 Division of Practice Advancement and Clinical Education, UNC Eshelman School of Pharmacy, University of North Carolina, North Carolina, Chapel Hill, United States of America; 2 Concept Foundation, Geneva, Switzerland; 3 Ghana Health Service, Accra, Ghana; 4 Department of Maternal and Child Health, UNC Gillings School of Global Public Health, University of North Carolina, North Carolina, Chapel Hill, United States of America; University of Cape Town, SOUTH AFRICA

## Abstract

Uterotonics are essential in preventing postpartum hemorrhage (PPH), the leading direct cause of maternal death worldwide. However, uterotonics are often substandard in low- and middle-income countries, contributing to poor maternal health outcomes. This study examines the health and economic impact of substandard uterotonics in Ghana. A decision-tree model was built to simulate vaginal and cesarean section births across health facilities, uterotonic quality and utilization, PPH risk and diagnosis, and resulting health and economic outcomes. We utilized delivery data from Ghana’s maternal health survey, risks of health outcomes from a Cochrane review, and E-MOTIVE trial data for health outcomes related to oxytocin quality. We compared scenarios with and without substandard uterotonics, as well as scenarios altering uterotonic use and care-seeking behaviors. We found that substandard uterotonic use contributes to $18.8 million in economic burden annually, including $6.3 million and $4.8 million in out-of-pocket expenditures in public and private sectors, respectively. Annually, the National Health Insurance Scheme bears $1.6 million in costs due to substandard uterotonic use. Substandard uterotonics contribute to $6 million in long-term productivity losses from maternal mortality annually. Improving the quality of uterotonics could reduce 20,000 (11%) PPH cases, 5,000 (11%) severe PPH cases, and 100 (11%) deaths due to PPH annually in Ghana. Ensuring the quality of uterotonics would result in millions of dollars in cost savings and improve maternal health outcomes for the government and families in Ghana. Cost savings from improving uterotonic quality would provide financial protection and help Ghana advance toward Universal Health Coverage.

## Introduction

Uterotonic medications reduce the risk of postpartum hemorrhage (PPH), the leading cause of direct maternal deaths worldwide. A third of global maternal deaths are attributed to obstetric hemorrhage [1, 2]. PPH affects 14 million women each year and leads to more than 70,000 maternal deaths annually [1, 3]. The majority of maternal deaths attributed to PPH (about 99%) occur in developing countries, with two-thirds of maternal deaths occurring in Sub-Saharan Africa [1]. The World Health Organization (WHO) recommends prophylactic uterotonics to prevent PPH during the third stage of labor [4, 5].

The problem is that uterotonics have been found to have some of the highest failure rates through quality testing among essential medicines in low- and middle-income countries (LMICs) [6–9]. A 2016 systematic review of oxytocin samples in LMICs found a high prevalence of substandard oxytocin especially in Sub-Saharan Africa [6]. A 2020 systematic review found that 75% of ergometrine samples and nearly 45% of oxytocin and misoprostol samples failed quality tests in LMICs [6, 7].

Substandard medicines fail to meet either their quality standards or specification, or both [10], as a result of poor manufacturing, shipping or storage conditions, or when the drug is sold beyond the expiration date. Substandard medicines are detrimental to health systems because they divert resources to ineffective therapies, which lead to medical complications and prolonged illnesses [8]. In addition to negative impacts on population health, substandard medicines negatively impact economies [11]. Consumers and health centers are burdened with the cost of resources wasted on ineffective treatments, and treating additional complications caused by substandard medicines [11, 12]. Governments, companies, and society as a whole are burdened with decreased economic productivity resulting from prolonged illnesses and reduced output and tax revenues [11, 12].

Despite the threat of substandard uterotonics on health outcomes, there are currently no studies that have estimated the resulting economic burden, which can lead to underinvestment in methods to prevent maternal mortality. One of the goals of Universal Health Coverage (UHC) is to protect patients from financial hardship that results from payment for medical care. By incorporating medicine quality assurance, the government can protect populations from the cost of additional care incurred by utilization of substandard medicines and extend UHC [13]. This case study focuses on Ghana, where substandard uterotonics and high maternal mortality have been documented [7, 14]. This study examines the health and economic impact of substandard uterotonics in Ghana examining the burden on the government, healthcare providers, and families.

## Materials and methods

### Decision tree model structure

**Fig 1:**
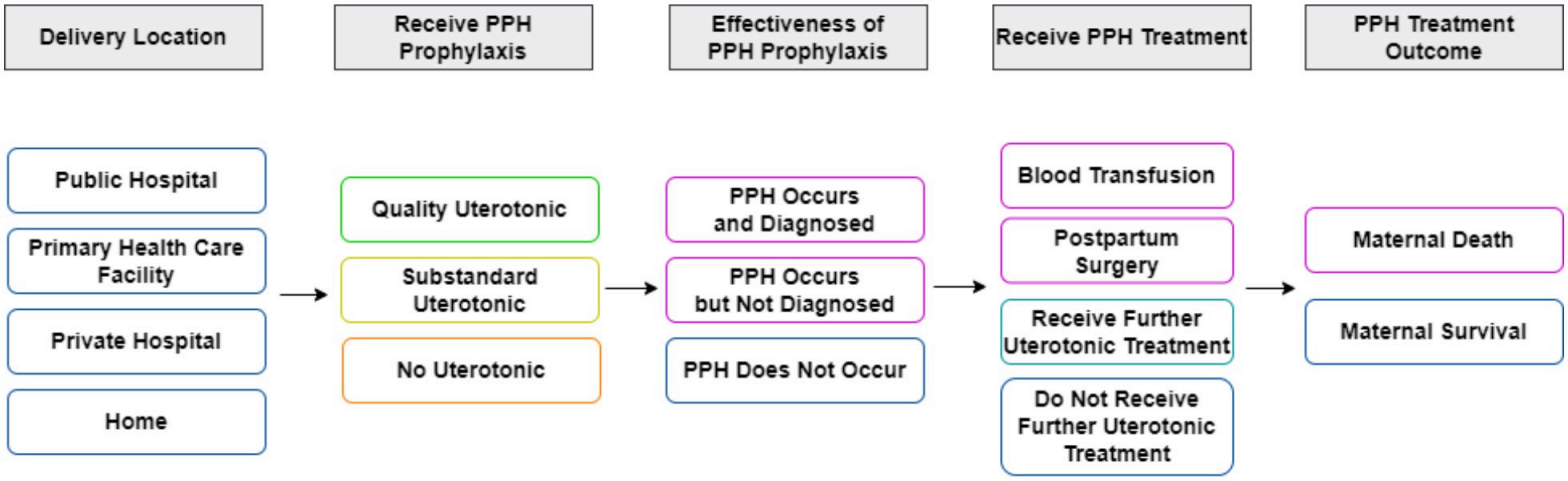
Model structure.

A decision tree model was built to demonstrate the impact of substandard uterotonics. Fig 1 presents the model structure, beginning with an annual estimate of the number of birthing mothers in Ghana. A detailed illustration of the decision tree model is included in S1 Fig. We simulated 100,000 mothers and multiplied the results to estimate population-level counts. Birthing mothers in the simulation faced a probability of a location of delivery (i.e. public hospital, public primary health care facility, private hospital, or home) and mode of delivery (i.e. vaginal or cesarean section). To capture some of the heterogeneity across the population, maternal background characteristics (i.e. age, region, urban/rural residence, wealth quintile and education) were incorporated to represent the distribution of birthing mothers across places and modes of delivery [15]. We simulated that for PPH prevention, some mothers received quality uterotonics, other mothers utilized substandard uterotonics, while the remainder of mothers did not receive prophylactic uterotonics. Those in the decision tree branches that received PPH prophylaxis received either oxytocin only, or oxytocin along with misoprostol. Based on the place/mode of delivery and uterotonic use, mothers faced the risk of PPH occurring, followed by the possibility of further treatment with uterotonics, blood transfusions, or postpartum surgery. Mothers with severe PPH faced the probability of death from PPH. The resulting health outcomes were summed throughout the model. Costs of care along with productivity losses due to maternal deaths were estimated. We followed the Consolidated Health Economic Evaluation Reporting Standards 2022 (CHEERS2022) checklist in reporting our study (S1 Table).

### Data inputs

We identified relevant literature including manuscripts, reports, and treatment guidelines to inform our model inputs. We collected data on utilization of uterotonics, their quality, and PPH associated costs in Ghana. Searches were conducted primarily through PubMed and supplemented by Google and ISI Web of Knowledge. Iterations of search terms such as “uterotonics,” “maternal mortality,” “postpartum hemorrhage,” “utilization,” “costs,” “quality,” and “Ghana” were applied. Institutional websites of the World Health Organization (WHO), United Nations (UN), United Nations Children’s Fund (UNICEF), the World Bank, Ghana Ministry of Health and National Medicine Regulatory Authority sites, and Ghana’s latest Maternal Health Survey (MHS) data were also utilized [15].

Table 1 presents key data inputs for the model. Birth rate and maternal mortality rate data was extracted from the UN population projections and WHO’s summary of trends in maternal mortality [14, 16]. Data for life expectancy and gross domestic product (GDP) per capita were abstracted from the World Bank [17, 18]. The proportion of severe PPH cases at home being referred to health facilities was assumed using data from the 2011 Ghana Emergency Obstetric and Newborn Care National Report [19]. Demographic information about women’s age, region of residence, wealth quintile, education, rurality, and National Health Insurance Scheme (NHIS) status was pulled from Ghana’s 2017 MHS [20]. The MHS data was also used to abstract the likelihood of women delivering at different facilities based on age, region, urban/rural residence, wealth quintile, education, and NHIS status. Oxytocin and misoprostol were modeled as these uterotonics were primarily utilized for prevention of PPH in Ghana [21]. Data from a recent Cochrane Review was used to calculate transition probabilities of PPH-related health outcomes [22]. PPH was defined as birthing mothers experiencing blood loss of 500 mL or more (PPH ≥500 ml), and severe PPH with blood loss of 1000 mL or more (PPH ≥1000 ml). Probabilities of health outcomes varied by modes of delivery (vaginal or c-section) and PPH prophylaxis regimens [22]. The baseline prevalence of substandard oxytocin in Ghana was extracted from a 2013 post-market quality surveillance study of oxytocin on the Ghanaian market conducted by the Ghana Food and Drugs Authority (FDA), Laboratory Services Department, and Promoting the Quality of Medicines Program [23].

**Table 1:** Key Ghana model data inputs.

Parameter variable	Unit	Value (Uncertainty range)					Source
**Demographics**							
Total population		32,833,031					UN
Birth rate	Per 1,000 people	29					UN [16]
Maternal mortality rate	Per 100,000 live births	263		UN			WHO [14]
Mean age of maternal death	Years	26.5					Gumanga [30]
Life expectancy at birth, female	Years	66					World Bank [18]
GDP per Capita	USD	2,363					World Bank [17]
**Population Characteristics**							
*Region and NHIS status*		Urban	Rural	NHIS Covered		NHIS Not Covered	
Western	%	66	34	45		55	MHS [20]
Central	%	82	18	39		61	
Greater Accra	%	92	8	36		64	
Volta	%	55	45	40		60	
Eastern	%	16	84	57		43	
Ashanti	%	82	18	41		59	
Brong Ahafo	%	61	39	54		46	
Norther	%	44	56	55		45	
Upper East	%	43	57	54		46	
Upper West	%	42	58	60		40	
*Wealth Quintile*		Urban		Rural			
Poorest	%	7		34			MHS [20]
Poorer	%	13		23			
Middle	%	17		19			
Richer	%	26		16			
Richest	%	36		8			
**Care-seeking locations and birth methods by mother’s characteristics**							
*Rurality*		Urban		Rural			
Public Hospital and Vaginal birth	%	42		29			MHS [20]
Public Hospital and C-section	%	13		7			
PHCF and Vaginal birth	%	17		26			
Private Hospital and Vaginal birth	%	12		5			
Private Hospital and C-section	%	3		1			
Home	%	15		31			
*Wealth Quintile*		Poorest	Poorer	Middle	Richer	Richest	
Public Hospital and Vaginal birth	%	20	34	43	47	43	MHS [20]
Public Hospital and C-section	%	4	7	10	13	19	
PHCF and Vaginal birth	%	28	24	23	17	11	
Private Hospital and Vaginal birth	%	3	5	7	11	18	
Private Hospital and C-section	%	0.3	1	1	2	6	
Home	%	44	30	17	10	3	
*NHIS Coverage Status*		Covered		Not Covered			
Public Hospital and Vaginal birth	%	40		34			MHS [20]
Public Hospital and C-section	%	12		8			
PHCF and Vaginal birth	%	22		20			
Private Hospital and Vaginal birth	%	7		9			
Private Hospital and C-section	%	2		2			
Home	%	17		26			
Proportion of diagnosis with PPH	%	44					Valdes et al. [28]
Proportion of referrals among severe PPH from home and PHCF	%	47					Ghana EmONC National Report [19]
**Utilization of Uterotonics**		Baseline					
Oxytocin	%	34					E-MOTIVE Trial [24]
Oxytocin plus Misoprostol	%	66					
No uterotonics given	%	0					
**Proportion of substandard uterotonics**							
		Public sectors		Private sectors			
Oxytocin	%	53		58			Karikari et al. [23]
Misoprostol	%	38		32			Torloni et al. [7]
**Oxytocin Dose**							
		Substandard oxytocin		Quality assured oxytocin			
10 IU		69.2%		99.6%			E-MOTIVE Trial [24]
20 IU		29.8%		0.0%			
30 IU		1.0%		0.4%			
**Risk of health outcomes**							
Oxytocin + Misoprostol		Vaginal birth		C section			
PPH ≥500ml		0.085 (0.071–0.105)		0.423 (0.35–0.519)			Gallos et al. [22]
PPH ≥1000ml		0.025 (0.02–0.029)		0.124 (0.088–0.129)			
Oxytocin		Vaginal birth		C section			
PPH ≥500ml		0.122 (0.11–0.134)		0.604 (0.544–0.664)			
PPH ≥1000ml		0.03 (0.027–0.033)		0.133 (0.12–0.146)			
Misoprostol		Vaginal birth		C-section			
PPH ≥500ml		0.157 (0.141–0.173)		0.132 (0.119–0.145)			
PPH ≥1000ml		0.044 (0.04–0.048)		0.036 (0.032–0.04)			
Carbetocin		Vaginal birth		C-section			
PPH ≥500ml		0.087 (0.078–0.096)		0.435 (0.392–0.479)			
PPH ≥1000ml		0.026 (0.023–0.029)		0.116 (0.104–0.128)			
No prophylactic uterotonics		Vaginal birth					
PPH ≥500ml		0.241 (0.193–0.289)					
PPH ≥1000ml		0.05 (0.038–0.058)					
Risk of PPH quality assured vs. substandard uterotonics							
PPH ≥500ml	Risk Ratio	1.29					E-MOTIVE Trial [24]
PPH ≥1000ml	Risk Ratio	1.26					
Proportion of receiving additional uterotonics treatment among diagnosed PPH							
quality assured uterotonics	%	70					Calibrated based on E-MOTIVE Trial [24]
substandard uterotonics	%	74					
Proportion of receiving additional uterotonics treatment among undiagnosed PPH							
quality assured uterotonics	%	35					Assumption
substandard uterotonics	%	37					
Proportion of blood transfusion among diagnosed PPH							
quality assured uterotonics	%	23					Calibrated based on E-MOTIVE Trial [24]
substandard uterotonics	%	39					
Proportion of blood transfusion among undiagnosed PPH							
quality assured uterotonics	%	11					Assumption
substandard uterotonics	%	20					
**Out-of-pocket costs to women and their families (2021 USD)**							
Public hospital							
Vaginal birth							KOL Opinion
No PPH	USD	172.24					
Mild PPH	USD	258.37					
Severe PPH without surgery	USD	516.73					
Severe PPH with surgery	USD	775.10					
C-section							
No PPH	USD	344.49					
Mild PPH	USD	430.61					
Severe PPH	USD	516.73					
PHCF							
Vaginal birth							
No PPH	USD	134.57					
Mild PPH	USD	201.85					
Severe PPH without surgery	USD	403.70					
Private hospital							
Vaginal birth							
No PPH	USD	595.70					
Mild PPH	USD	975.38					
Severe PPH without surgery	USD	1,621.29					
Severe PPH with surgery	USD	2,879.30					
C-section							
No PPH	USD	1,242.60					
Mild PPH	USD	1,622.28					
Severe PPH	USD	1,837.58					
**Costs to National Health Insurance Scheme (2021 USD)**							
Vaginal birth							
No PPH	USD	66.03					National Health Insurance Authority [29]
Mild PPH	USD	131.78					
Severe PPH without surgery	USD	131.78					
Severe PPH with surgery	USD	376.61					
C-section							
No PPH	USD	152.55					
Mild PPH	USD	218.29					
Severe PPH	USD	218.29					

C-section = Caesarean section; IU = international unit; KOL = Key Opinion Leader; MHS = Maternal Health Survey; PHCF = Primary health care facility; PPH = postpartum hemorrhage; UN = United Nations; USD = United States dollars; WHO = World Health Organization.

Data relating PPH outcomes with oxytocin quality and uterotonic utilization were extracted from the E-MOTIVE trial [24–26]. The E-MOTIVE trial is a randomized multicenter trial that examines the early detection of PPH and treatment using a calibrated blood-collection drape and a bundle of first response treatments in four LMICs [25–27]. The trial estimated the risk of PPH ≥500 ml and PPH ≥1000 ml occurring among mothers delivering at facilities that utilize quality and substandard uterotonics [24–26]. To estimate additional treatments and blood transfusions resulting from PPH, we calibrated the model using data collected from the E-MOTIVE trial [24–26, 28]. To do this, we ran the model 1,000 times using a range of inputs for the risk of each outcome, and chose the inputs that came closest to the rates seen in the E-MOTIVE trial [24, 26]. Our resulting inputs produced outputs that are within our target threshold of 10%. The number of doses of oxytocin used per patient in facilities that utilized quality oxytocin and those with substandard oxytocin were abstracted from the E-MOTIVE trial [24–26]. Differences were found between reported and projected cases of PPH due to trends in care seeking and reporting [28], so inputs were classified based on whether the PPH cases were diagnosed or not.

Key opinion leaders from Ghana provided information regarding the utilization of uterotonics, current practice on maternal care, and healthcare expenditures related to PPH that were unable to be retrieved from the literature. The names of key opinion leaders are noted in the acknowledgements. Additionally, we collected data on the costs incurred by NHIS from the National Health Insurance Authority report on NHIS tariffs paid to tertiary hospitals to treat PPH cases [29]. The baseline model was validated against existing data on PPH cases and deaths [20, 25].

### Model outputs

The primary model outputs are estimates of the health impact, economic impact, and UHC outcomes attributable to the use of substandard uterotonics in the third stage of labor. The number of PPH, severe PPH, additional uterotonic treatment, blood transfusions, maternal deaths, and years of life lost (YLL) due to PPH are reported. The economic impact (in 2021 $USD) includes estimates of the overall annual burden of PPH, out-of-pocket (OOP) costs in public sectors, OOP costs in private sectors, costs borne by NHIS, and long-term productivity losses. OOP costs were estimated from hospital charges, including charges for medications, blood transfusions, and other surgery procedures. These hospital charges informed the estimates of the expenses that patients were paying or being charged. Productivity losses were estimated using years of life lost from maternal death from PPH multiplied by the annual GDP per capita discounted at 3% [30]. UHC related outcomes from the perspectives of families, healthcare providers, and governments were estimated. For families, these outcomes included the number of mothers receiving substandard uterotonics, number of cases of PPH and severe PPH receiving substandard uterotonics, and number of maternal deaths due to PPH that could be averted by using quality uterotonics. UHC outcomes for healthcare providers included estimates of additional oxytocin doses saved and numbers of blood transfusions averted by using quality uterotonics. UHC outcomes for payors referred to costs from additional treatments, blood transfusions, and other related commodities due to substandard uterotonics that are paid for by insurance from NHIS. UHC outcomes for the government included the percent of cases of PPH receiving substandard uterotonics, percent of cases of severe PPH receiving substandard uterotonics, and percent of maternal deaths due to PPH averted by using quality uterotonics.

### Model scenarios

Scenario analyses were conducted to estimate the impact of uterotonic quality and the impact of utilizing different uterotonics. We first simulated a baseline scenario that uses the reported prevalence of substandard uterotonics and compared it with a scenario where all uterotonics are of adequate quality. To examine the impact of the choice of uterotonic, we simulated a scenario where facility births all utilize quality oxytocin alone, as well as a scenario where facility births all utilize quality oxytocin and misoprostol together. We examined the impact of care-seeking and uterotonic quality together by running a scenario where all women use facility care and utilize quality uterotonics, compared to a scenario where all women use facility care and utilize substandard uterotonics. Finally, we examined a scenario where facility births all utilize quality heat-stable carbetocin. We compared these results by assessing the difference between the baseline scenario and the remaining six scenarios.

### Sensitivity analysis

We conducted sensitivity analyses where key input parameters were ranged probabilistically across model runs to account for uncertainties in epidemiological and cost inputs. In each run, values of key inputs were drawn from uncertainty ranges. Inputs for risks of health outcomes and quality of uterotonics were varied based on beta distributions, while cost data were ranged using gamma distributions. Parameters that were varied during the probabilistic sensitivity analysis along with their uncertainty ranges are listed in Table 1. All outcomes are based on an average of 1,000 simulation runs in the model.

## Results

### Annual PPH burden in Ghana

Based on an estimate of 919,325 pregnant women delivering in Ghana per year, our model simulated 182,450 (IQR 177,657–187,030) cases of PPH including 45,273 (43,675–46,711) severe cases of PPH annually. Of those cases, only 61,679 (59,319–63,934) PPH cases including 17,753 (17,099–18,380) severe PPH cases were estimated to be diagnosed per year. This resulted in an estimate of 971 (901–1,039) maternal deaths that were attributed to PPH annually in Ghana. Additional uterotonic treatments for PPH were given to 72,857 (70,696–74,999) cases and blood transfusions were estimated to be required in 32,812 (31,730–33,797) PPH cases per year. The total economic burden of childbirths with PPH in Ghana was estimated at $143.1 million (~GH₵ 830.8 million [31]; $136.4–$150.0 million) annually, which consisted of about $46.0 million (~GH₵267.1 million; $42.2–$49.5 million) in out-of-pocket expenditures at public sectors, $31.1 million (~GH₵180.6 million; $27.8–$34.1 million) at private sectors, and $11.7 million (~GH₵67.9 million; $11.3–$12.0 million) borne by NHIS. Costs due to long-term productivity losses were estimated at $54.3 million (~GH₵315.2 million; $50.4–$58.1 million) from annual maternal deaths due to PPH.

### Impact of substandard uterotonics

Table 2 presents the annual health and economic impact of substandard uterotonics in Ghana. Our model estimated that substandard uterotonics were responsible for an estimated 107 (28–193) annual deaths and 2,539 YLL (652–4,566) due to PPH. We estimated that annually $18.8 million (~GH₵109.1million; $13.5-$23.8 million) in total economic burden to treat PPH could be attributed to substandard uterotonics. Annual out-of-pocket expenditures due to substandard uterotonics were $6.3 million (~GH₵36.6 million; $5.4-$7.2 million) in public sectors and $4.8 million (~GH₵27.9 million; $3.9-$5.7 million) in private sectors. Substandard uterotonic use was responsible for $1.6 million (~GH₵9.3 million; $1.4-$1.8 million) in annual NHIS costs and $6.0 million (~GH₵34.8 million; $1.5-$10.8 million) in long-term productivity losses due to maternal deaths. Our model also estimated that 20,035 (17,548–22,202) PPH including 4,955 (4,027–5,819) severe PPH cases are attributed to substandard uterotonic use annually. Of those cases, substandard uterotonics contributed to 8,837 (7,253–10,317) diagnosed PPH cases including 2,191 (1,701–2,620) diagnosed severe PPH cases. The number of cases requiring additional uterotonic treatment and number of cases requiring blood transfusions due to substandard uterotonics were estimated to be 12,506 (11,151–13,785) and 12,518 (11,721–13,275) respectively, per year. S2 Fig illustrates the range around the benefits of improving the quality of uterotonics in reducing PPH cases and resulting in total cost savings through a probabilistic sensitivity analysis.

**Table 2:** Annual health and economic burden of postpartum hemorrhage due to substandard uterotonics in Ghana.

	Baseline	IQR	No Substandard Uterotonics	Difference	IQR of difference	Diff%
**PPH ≥500ml**	182,450	177,657–187,030	162,415	-20,035	-22,202—-17,548	-11%
**PPH ≥1000ml**	45,273	43,675–46,711	40,318	-4,955	-5,819—-4,027	-11%
**PPH ≥500ml diagnosed**	61,679	59,319–63,934	52,842	-8,837	-10,317—-7,253	-14%
**PPH ≥1000ml diagnosed**	17,753	17,099–18,380	15,562	-2,191	-2,620—- 1,701	-12%
**Number of additional uterotonic treatment**	72,857	70,696–74,999	60,351	-12,506	-13,785—-11,151	-17%
**Number of blood transfusions**	32,812	31,730–33,797	20,293	-12,518	-13,275—-11,721	-38%
**Number of deaths due to PPH**	971	901–1,039	864	-107	-193—-28	-11%
**Total economic burden of PPH**	$143,060,554	$136,385,117—$149,708,565	$124,301,638	-$18,758,916	-$23,798,210—-$13,457,218	-13%
**Total OOP costs public**	$46,016,016	$42,203,341—$49,535,409	$39,685,101	-$6,330,914	-$7,167,389—-$5,355,842	-14%
**Total OOP costs private**	$31,083,160	$27,769,019—$34,140,614	$26,273,157	-$4,810,002	-$5,660,464—-$3,920,236	-15%
**Total NHIS costs**	$11,693,937	$11,347,062—$12,030,008	$10,077,334	-$1,616,603	-$1,813,604—-$1,388,895	-14%
**Long term productivity loss**	$54,267,442	$50,358,492–$58,066,425	$48,266,046	-$6,001,396	-$10,791,106—-$1,541,587	-11%

Diff = difference; IQR = Interquartile range; NHIS = National Health Insurance Scheme; OOP: out-of-pocket; PPH: postpartum hemorrhage.

The costs of substandard uterotonics that are borne by different stakeholders in Ghana as presented in Table 3. We estimated that annually 354,428 mothers receive substandard uterotonics. Moreover, 77,360 of the 182,450 total PPH cases (42%) including 20,155 of the 45,273 severe PPH cases (45%) were mothers that received substandard uterotonics. With quality uterotonics, healthcare providers would administer a total of 109,893 fewer doses of oxytocin and handle 12,518 fewer cases of blood transfusions annually. The model simulated that 11% of the deaths due to PPH could be averted by using quality uterotonics.

**Table 3:** Annual impact of substandard uterotonics in Ghana by perspective.

Perspective	Description	Estimate
Families	No. of mothers receiving substandard uterotonics	354,428
	No. of cases of postpartum hemorrhage receiving substandard uterotonics	77,360
	No. of cases of severe postpartum hemorrhage receiving substandard uterotonics	20,155
	Out-of-pocket costs from additional treatments, blood transfusions, and longer hospitalizations due to substandard uterotonics (USD)	$11,140,917
	No. of maternal deaths averted by using quality uterotonics	107
	No. of years of life saved by using quality uterotonics	2,539
Healthcare Providers	No. of doses of oxytocin saved by using quality uterotonics	109,893
	No. of blood transfusions averted by using quality uterotonics	12,518
Payors	Payor costs from additional treatments, blood transfusions, other related commodities due to substandard uterotonics (USD)	$1,616,603
Governments	% receiving substandard uterotonics among postpartum hemorrhage cases	42%
	% receiving substandard uterotonics among severe postpartum hemorrhage	45%
	% of maternal deaths averted by using quality uterotonics	11%

No. = Number; USD = United States dollars.

Table 4 describes the burden of substandard uterotonics across the population in Ghana. The outcomes of PPH ≥500 ml, PPH ≥1000 ml, maternal death by PPH, total economic burden of PPH, and OOP costs are presented based on rurality, wealth quintile, and NHIS status. Our analysis shows that all categories of families, regardless of domicile, wealth, or insurance status, are impacted by substandard uterotonics in Ghana. For example, eliminating substandard uterotonics would equally benefit both mothers covered and not covered by NHIS. For women who gave birth and were covered by NHIS, the total economic burden was $74.7 million (~GH₵433.7 million) annually, with $10.0 million (~GH₵58.1 million; 13%) attributable to substandard uterotonic use. For those without NHIS coverage, baseline total economic burden was $68.4 million (~GH₵397.1 million), with $8.8 million (~GH₵51.1 million; 13%) due to substandard uterotonic use. For those who were covered by NHIS, there were 87,054 PPH cases, where 10,259 (12%) PPH cases could be prevented by using quality uterotonics. For the 95,396 PPH cases that were not covered by NHIS, 9,775 (10%) cases could be prevented by using quality uterotonics.

**Table 4:** Annual burden of substandard uterotonics in Ghana by population characteristics (rurality, wealth, and insurance status).

	Baseline	No Substandard Uterotonics	Difference	Proportion of reduction	Difference % from baseline
**PPH ≥500 ml**					
Overall	182,450	162,415	-20,035		-11%
Urban	104,362	91,659	-12,703	63%	-12%
Rural	78,088	70,755	-7,332	37%	-9%
Poorest	32,003	30,159	-1,844	9%	-6%
Poorer	30,056	27,526	-2,529	13%	-8%
Middle	30,060	26,758	-3,302	16%	-11%
Richer	38,099	33,293	-4,806	24%	-13%
Richest	52,232	44,679	-7,553	38%	-14%
NHIS	87,054	76,794	-10,259	51%	-12%
No NHIS	95,396	85,620	-9,775	49%	-10%
**PPH ≥1000 ml**					
Overall	45,273	40,318	-4,955		-11%
Urban	27,933	24,592	-3,341	67%	-12%
Rural	17,340	15,726	-1,613	33%	-9%
Poorest	7,258	6,781	-476	10%	-7%
Poorer	7,148	6,539	-608	12%	-9%
Middle	7,502	6,676	-826	17%	-11%
Richer	9,724	8,536	-1,188	24%	-12%
Richest	13,641	11,785	-1,856	37%	-14%
NHIS	21,890	19,361	-2,528	51%	-12%
No NHIS	23,383	20,957	-2,426	49%	-10%
**Deaths due to PPH**					
Overall	971	864	-107		-11%
Urban	597	529	-69	64%	-11%
Rural	373	335	-39	36%	-10%
Poorest	157	144	-13	12%	-8%
Poorer	153	139	-13	12%	-9%
Middle	161	144	-17	16%	-11%
Richer	209	185	-25	23%	-12%
Richest	292	252	-40	37%	-14%
NHIS	469	414	-55	51%	-12%
No NHIS	502	450	-53	49%	-10%
**Total economic burden of PPH**					
Overall	$143,060,554	$124,301,638	-18,758,916		-13%
Urban	$95,851,691	$82,865,454	-12,986,237	69%	-14%
Rural	$47,208,863	$41,436,185	-5,772,679	31%	-12%
Poorest	$15,923,043	$14,341,117	-1,581,926	8%	-10%
Poorer	$18,508,467	$16,432,690	-2,075,778	11%	-11%
Middle	$21,675,663	$18,937,823	-2,737,840	15%	-13%
Richer	$31,906,629	$27,587,124	-4,319,505	23%	-14%
Richest	$55,046,752	$47,002,885	-8,043,868	43%	-15%
NHIS	$74,661,408	$64,660,455	-10,000,953	53%	-13%
No NHIS	$68,399,146	$59,641,184	-8,757,963	47%	-13%
**Total OOP costs**					
Overall	$77,099,175	$65,958,259	-11,140,917		-14%
Urban	$55,376,630	$47,228,568	-8,148,062	73%	-15%
Rural	$21,722,546	$18,729,691	-2,992,855	27%	-14%
Poorest	$5,670,184	$4,982,445	-687,739	6%	-12%
Poorer	$8,246,988	$7,139,515	-1,107,473	10%	-13%
Middle	$10,623,836	$9,117,106	-1,506,730	14%	-14%
Richer	$17,525,299	$14,962,689	-2,562,611	23%	-15%
Richest	$35,032,869	$29,756,504	-5,276,365	47%	-15%
NHIS	$36,768,722	$31,442,880	-5,325,843	48%	-14%
No NHIS	$40,330,453	$34,515,379	-5,815,074	52%	-14%
**NHIS costs**					
Overall	$11,693,937	$10,077,334	-1,616,603		-14%
Urban	$7,081,728	$6,080,048	-1,001,680	62%	-14%
Rural	$4,612,209	$3,997,286	-614,922	38%	-13%
Poorest	$1,493,564	$1,317,756	-175,808	11%	-12%
Poorer	$1,734,965	$1,504,566	-230,399	14%	-13%
Middle	$2,061,808	$1,776,719	-285,090	18%	-14%
Richer	$2,690,451	$2,307,625	-382,827	24%	-14%
Richest	$3,713,148	$3,170,668	-542,480	34%	-15%
NHIS	$11,693,937	$10,077,334	-1,616,603	100%	-14%
No NHIS	$0	$0	0	-	-
**Long term productivity losses**					
Overall	$54,267,442	$48,266,046	-6,001,396		-11%
Urban	$33,393,333	$29,556,838	-3,836,495	64%	-11%
Rural	$20,874,109	$18,709,208	-2,164,901	36%	-10%
Poorest	$8,759,295	$8,040,915	-718,379	12%	-8%
Poorer	$8,526,515	$7,788,609	-737,906	12%	-9%
Middle	$8,990,019	$8,043,998	-946,020	16%	-11%
Richer	$11,690,878	$10,316,811	-1,374,067	23%	-12%
Richest	$16,300,736	$14,075,712	-2,225,023	37%	-14%
NHIS	$26,198,749	$23,140,241	-3,058,508	51%	-12%
No NHIS	$28,068,693	$25,125,805	-2,942,889	49%	-10%

NHIS = National Health Insurance Scheme; OOP = Out-of-pocket; PPH = Postpartum hemorrhage.

When it comes to wealth quintile, women from the poorest to the richest categories would experience a reduction in economic burden of PPH ranging from 10% to 15% by improving the quality of uterotonics. Baseline economic burden of PPH was $15.9 million (~GH₵92.3 million) for the poorest population, $18.5 million (~GH₵107.4 million) for the poorer, $21.7 million (~GH₵126 million) for the middle, $31.9 million (~GH₵185.2 million) for the richer, and $55.0 million (~GH₵319.3 million) for the richest quintile. Cost savings by avoiding substandard uterotonic use was estimated at $1.6 million (~GH₵9.3 million; 10%) for the poorest population, $2.1 million (~GH₵12.2 million; 11%) for the poorer, $2.7 million ((~GH₵15.7 million; 13%) for the middle, $4.3 million (~GH₵25 million; 14%) for the richer, and $8 million (~GH₵46.4 million; 15%) for the richest quintile. Baseline PPH counts by wealth quintile was 32,003 for those considered poorest, 30,056 for poorer, 30,060 for middle, 38,099 for richer, and 52,323 for richest. Substandard uterotonic use accounted for 1,844 (6%) PPH cases among birthing women considered poorest, 2,529 (8%) for poorer, 3,302 (11%) for middle, 4,806 (13%) for richer, and 7,553 (14%) for richest quintile.

Due to higher levels of care-seeking in urban areas, ensuring the quality of uterotonics would result in a 14% reduction in urban economic burden of PPH, and 12% reduction in rural economic burden in Ghana. The total economic burden of PPH was estimated at $95.8 million (~GH₵556.2 million) for women giving birth in urban areas and $47.2 million (~GH₵274 million) for women in rural areas. Annually, substandard uterotonic use accounted for $13.0 million (~GH₵75.5 million; 14%) in urban and $5.8 million (~GH₵33.7 million;12%) in rural areas in avertable healthcare expenditures and productivity losses. The prevalence of PPH (PPH ≥500 ml) was estimated at 104,362 cases among women giving birth in urban areas, compared to 78,088 cases in rural areas annually. By ensuring the quality of uterotonics, 12,703 (12%) PPH cases in urban areas and 7,332 (9%) PPH cases in rural areas could be prevented.

### Scenario results

Table 5 shows the annual health and economic outcomes for each modeled scenario. We first ranged our assumption of uterotonic use, where we simulated scenarios of all facility births using oxytocin alone or using oxytocin and misoprostol in combination. When facility births were simulated to use quality oxytocin alone, annual severe PPH cases decreased by 2,162 (5%) cases, cases needing blood transfusion decreased by 7,798 (24%), maternal deaths decreased by 47 (5%), YLL decreased by 1,104 (5%), and long-term productivity loss decreased by $2.6 million (~GH₵15.1 million; 5%). Nevertheless, compared to the baseline where 66% of facility births are assumed to use oxytocin and misoprostol together, use of oxytocin alone is less effective. Therefore, cases of overall PPH increased by 8,651 (5%), cases needing additional uterotonic treatment increased by 1,851 (3%), overall economic burden increased by $1.6 million (~GH₵9.3 million; 5%), and total OOP costs increased by $3.5 million (~GH₵20.3 million; 5%). When facility births all used quality oxytocin and misoprostol, 34,595 (19%) overall PPH cases including 6,365 (14%) severe PPH cases,19,792 (27%) cases of additional uterotonics, 14,898 (45%) cases of blood transfusions, 143 (15%) maternal deaths, and 3,381 (15%) YLL were prevented annually. This led to a reduction in economic burden by $29.3 million (~GH₵170.1 million; 21%), total OOP costs by $18.6 million (~GH₵108 million; 24%), and long-term productivity loss by $8.0 million (~GH₵46.4 million; 15%) per year.

**Table 5:** Model scenario results.

	PPH ≥500 ml	PPH ≥1000 ml	Additional uterotonic treatment	Blood transfusions	Deaths	YLL	Total economic burden by PPH			
							Overall	Total OOP costs	Total NHIS costs	Long term productivity losses
**Baseline**	182,450	45,273	72,857	32,812	971	22,963	$143,060,554	$77,099,175	$11,693,937	$54,267,442
**No substandard uterotonics**	162,415	40,318	60,351	20,293	864	20,423	$124,301,638	$65,958,259	$10,077,334	$48,266,046
Difference	-20,035	-4,955	-12,506	-12,518	-107	-2,539	-18,758,916	-$11,140,917	-$1,388,895	-$6,001,396
Difference %	-11%	-11%	-17%	-38%	-11%	-11%	-13%	-14%	-14%	-11%
**Facility births all utilize quality Oxytocin**	191,101	43,110	74,708	25,014	924	21,858	$144,650,747	$80,612,799	$12,379,899	$51,658,050
Difference	8,651	-2,162	1,851	-7,798	-47	-1,104	$1,590,193	$3,513,623	$685,962	-$2,609,392
Difference %	5%	-5%	3%	-24%	-5%	-5%	5%	5%	6%	-5%
**Facility births all use quality Oxytocin + Misoprostol**	147,855	38,907	53,065	17,914	828	19,852	$113,723,921	$58,543,384	$8,902,624	$46,277,913
Difference	-34,595	-6,365	-19,792	-14,898	-143	-3,381	-$29,336,633	-$18,555,792	-$2,791,313	-$7,989,529
Difference %	-19%	-14%	-27%	-45%	-15%	-15%	-21%	-24%	-24%	-15%
**Facility births all utilize quality Carbetocin**	150,197	38,671	54,281	18,305	827	19,562	$115,215,200	$59,861,928	$9,121,607	$46,231,665
Difference	-32,253	-6,602	-18,576	-14,507	-144	-3,400	-$27,845,354	-$17,237,247	-$2,572,330	-$8,035,777
Difference %	-18%	-15%	-25%	-44%	-15%	-15%	-19%	-22%	-22%	-15%
**All facility births (quality)**	140,258	37,589	97,629	32,099	808	19,105	$127,180,509	$71,109,800	$10,919,182	$45,151,527
Difference	-42,192	-7,683	24,772	-713	-163	-3,857	-$15,880,045	-$5,989,375	-$774,755	-$9,115,915
Difference %	-23%	-17%	34%	-2%	-17%	-17%	-11%	-8%	-7%	-17%
**All facility births (substandard)**	189,226	49,780	140,025	74,565	1,073	25,382	$170,448,084	$95,731,852	$14,730,018	$59,986,214
Difference	6,777	4,507	67,168	41,753	102	2,420	$27,387,530	$18,632,677	$3,036,081	$5,718,772
Difference %	4%	10%	92%	127%	11%	11%	19%	24%	26%	11%

NHIS = National Health Insurance Scheme; OOP = Out-of-pocket; PPH = Postpartum hemorrhage; YLL = Years of life lost. The model scenarios are: 1) baseline scenario using reported uterotonic quality data; 2) scenario where all births do not utilize substandard uterotonics (all uterotonics are quality-assured); 3) scenario where all births taking place in facilities utilize quality-assured oxytocin; 4) scenario where all births taking place in facilities utilize quality-assured oxytocin and misoprostol; 5) scenario where all births taking place in facilities utilize quality-assured carbetocin; 6) scenario where all births taking place in facilities utilize all quality-assured uterotonics; 7) scenario where all births taking place in facilities utilize uterotonics of substandard quality.

We then examined a scenario where facility births used quality heat-stable carbetocin instead of oxytocin alone or oxytocin plus misoprostol. In this scenario, 32,253 (18%) PPH cases including 6,602 (15%) severe PPH cases, 18,576 (25%) cases of additional uterotonics treatment, 14,507 (44%) cases of blood transfusions, 144 (15%) maternal deaths, and 3,400 (15%) YLL could be averted annually. Annual cost savings for this scenario were $27.8 million (~GH₵161.4 million; 19%) in avertable economic burden, including $17.2 million (~GH₵99.9 million; 22%) in total OOP costs, and $8.0 million (~GH₵46.4 million; 15%) in long-term productivity losses.

In the scenario where all mothers delivered at facilities and were given quality uterotonics for PPH prevention, the model estimated that 42,192 (23%) PPH cases including 7,683 (17%) severe PPH, 713 (2%) cases needing blood transfusions, 163 (17%) maternal deaths, and 3,857 (17%) YLL could be avoided annually. However, complete use of health facilities and quality uterotonics requires additional uterotonic treatments among 24,772 cases (34%) per year. Yearly cost savings of $15.9 million (~GH₵92.3 million; 11%) in overall economic burden including $6.0 million (~GH₵34.8 million; 8%) in total OOP costs, and $9.1 million (~GH₵52.8 million; 17%) in long-term productivity losses were estimated from increased care-seeking and use of quality uterotonics. When all births were simulated in a facility and used substandard uterotonics, our model showed an annual increase in total PPH cases by 6,777 (4%), severe PPH by 4,507 (10%), cases of additional uterotonic treatment by 67,168 (92%), cases of blood transfusion by 41,753 (127%), maternal deaths by 102 (11%), and YLL by 2,420 (11%). Economic impacts showed an annual increase of $27.4 million (~GH₵159.1 million; 19%) in overall economic burden, including $18.6 million (~GH₵108 million; 24%) in total OOP costs, and $5.7 million (~GH₵33.1 million; 11%) in long-term productivity losses in a scenario with all births taking place at facilities and using substandard uterotonics.

## Discussion

This study is the first to demonstrate the economic impact of substandard uterotonics on population health and estimate the annual cost burden of substandard uterotonics on governments, healthcare providers, and families. This study shows that ensuring the quality of uterotonics in Ghana would not only improve maternal health outcomes but also substantially reduce costs to the government and families by reducing the number of PPH cases and other outcomes associated with PPH. Quality uterotonics would not only reduce 20,000 PPH and almost 5,000 severe PPH cases annually in Ghana, but also result in less healthcare resource use from reduced doses of uterotonic treatment and fewer blood transfusions. Moreover, use of quality uterotonics would reduce the leading direct cause of maternal death in Ghana by 11%.

Improving the quality of uterotonics would save millions of dollars annually in costs of PPH. These savings come from reduced OOP costs, savings to the NHIS, and reduced long-term productivity losses. These cost savings that could result from ensuring uterotonic quality would be better utilized elsewhere, for example funneled towards extending UHC so that more individuals could be covered by NHIS, more services could be covered by NHIS, and fewer individuals would become poor by paying healthcare expenditures. Within the scope of achieving UHC, the benefits of improving medicine quality have not been thoroughly discussed or investigated. This study provides evidence to make a case that both individuals and governments can benefit economically from improving uterotonic quality. To facilitate data collection for future studies, we included a table of suggested model inputs for other countries to also estimate the burden of substandard uterotonics (S2 Table). Taking steps to ensure medicine quality should be a priority within plans to expand UHC.

Common drivers of substandard medicines in LMICs are issues with pharmaceutical governance, weak technical capacity, and inadequate supply-chain management [32]. Since oxytocin is heat-sensitive, the WHO recommends that oxytocin be kept in the cold chain of 2–8 °C (35–46 °F) at all points during transport, distribution, and storage to ensure quality and efficacy [33]. Consistent product management for uterotonics is needed to ensure that mothers are not receiving substandard medicines and to reduce the health and economic burden of PPH. It is the collective responsibility of supply chain managers, warehousers, distributors, healthcare providers, and medicine regulators to ensure cold chain maintenance, and the responsibility of manufacturers to ensure proper storage labeling requirements [3]. Country policies and practices are also need to ensure that procurement of oxytocin meets quality requirements established by WHO or a regulatory authority recognized by the WHO [3]. National medicines regulatory authorities and ministries of health need to be bolstered to regulate the quality of uterotonics and ensure the systems are in place to maintain their quality.

Another opportunity for countries to reduce the burden of PPH would be a switch to using heat-stable carbetocin. Heat-stable carbetocin is another uterotonic that can be used for PPH prevention, but importantly, it does not require cold-chain storage and transportation, which can be advantageous in low-resource settings [34]. Heat-stable carbetocin has shown similar efficacy and adverse events profile to oxytocin within the CHAMPION trial [34, 35]. Moreover, in a meta-analysis, heat-stable carbetocin has been found to have similar safety and superior effectiveness compared to oxytocin [36]. Unfortunately, heat-stable carbetocin is not widely available and costs slightly more per dose than oxytocin [35]. In terms of health economic evidence, a network meta-analysis and systematic review found the relative cost-effectiveness of heat-stable carbetocin compared to alternative strategies to be inconclusive due to the uncertainty and inconsistency in the data reported on adverse events [37, 38]. Yet some recent studies in LMICs have shown that heat-stable carbetocin can be a cost-effective alternative to oxytocin [39–41]. The manufacturer of heat-stable carbetocin has made a commitment to increase its availability to primary health care facilities in LMICs at an affordable price [35]. If made financially viable, utilization of heat-stable carbetocin could offer an alternative to oxytocin and misoprostol that avoids the cold chain entirely and could be a sustainable option.

As with all modeling studies, this study has limitations including those related to quality and availability of data included in the model. There were gaps in available data regarding utilization of uterotonics and OOP costs for women and their families across Ghana. To address this, the team utilized key informants vetted by a steering committee to advise on the data inputs for uterotonic utilization and costs, and provide insights about data assumptions. The model was also calibrated and validated against existing data. Secondly, the relationship of substandard uterotonics to PPH, further treatment, and death are not well studied. Data from the recent E-MOTIVE trial from Nigeria were used in this study, with the assumption that the relationship of quality to outcomes is similar across settings [24, 26]. Thirdly, there were limited data to account for the population heterogeneity across health and economic outcomes. Sensitivity analyses and a wide variety of scenario analyses were conducted to provide a range around the inputs and compare the impact of improving the quality of uterotonics to other potential uterotonic policy options.

Substandard uterotonics cause avoidable harm to mothers while also burdening the health system, NHIS, and families with preventable costs. This study provides evidence for policymakers to act on the issue of substandard uterotonics to reduce maternal morbidity and mortality. A broad range of stakeholders, including families, providers, payors, and the government, would all benefit from reducing availability of, and access to, substandard uterotonics. Enhancing the quality of uterotonics would result in cost savings which would provide financial protection for health and opportunities to extend NHIS coverage to advance UHC in Ghana.

## Supporting information

S1 Fig Diagrammatic depiction of the decision tree model. (DOCX)

S2 Fig Probabilistic sensitivity analysis. Probabilistic Sensitivity Analysis was conducted for the 1,000 simulations to show annual cost savings versus total PPH case reduction by improving quality of uterotonics. The orange data point represents the baseline estimate while the blue data points are the remaining simulated estimates.
(DOCX)

S1 Table Consolidated Health Economic Evaluation Reporting Standards 2022 (CHEERS2022) statement: Updated reporting guidance for health economic evaluations. From: Husereau D, Drummond M, Augustovski F, et al. Consolidated Health Economic Evaluation Reporting Standards 2022 (CHEERS 2022) Explanation and Elaboration: A Report of the ISPOR CHEERS II Good Practices Task Force. Value Health 2022;25. doi:10.1016/j.jval.2021.10.008.
(DOCX)

S2 Table Suggested model input data to estimate burden of substandard uterotonics. C-section = Caesarean section; DHS = Demographic and Health Survey; KOL = Key Opinion Leader; PPH = postpartum hemorrhage; USD = United States dollars; WHO = World Health Organization.
(DOCX)
