# Supplementary figures and images for: Modeling the economic burden of postpartum hemorrhage due to substandard uterotonics in Ghana
Source: PLOS Glob Public Health. 2024 Jun 20;4(6):e0003181. doi: 10.1371/journal.pgph.0003181 (PMC11189185; doi:10.1371/journal.pgph.0003181)

S1 Fig. Diagrammatic Depiction of the Decision Tree Model


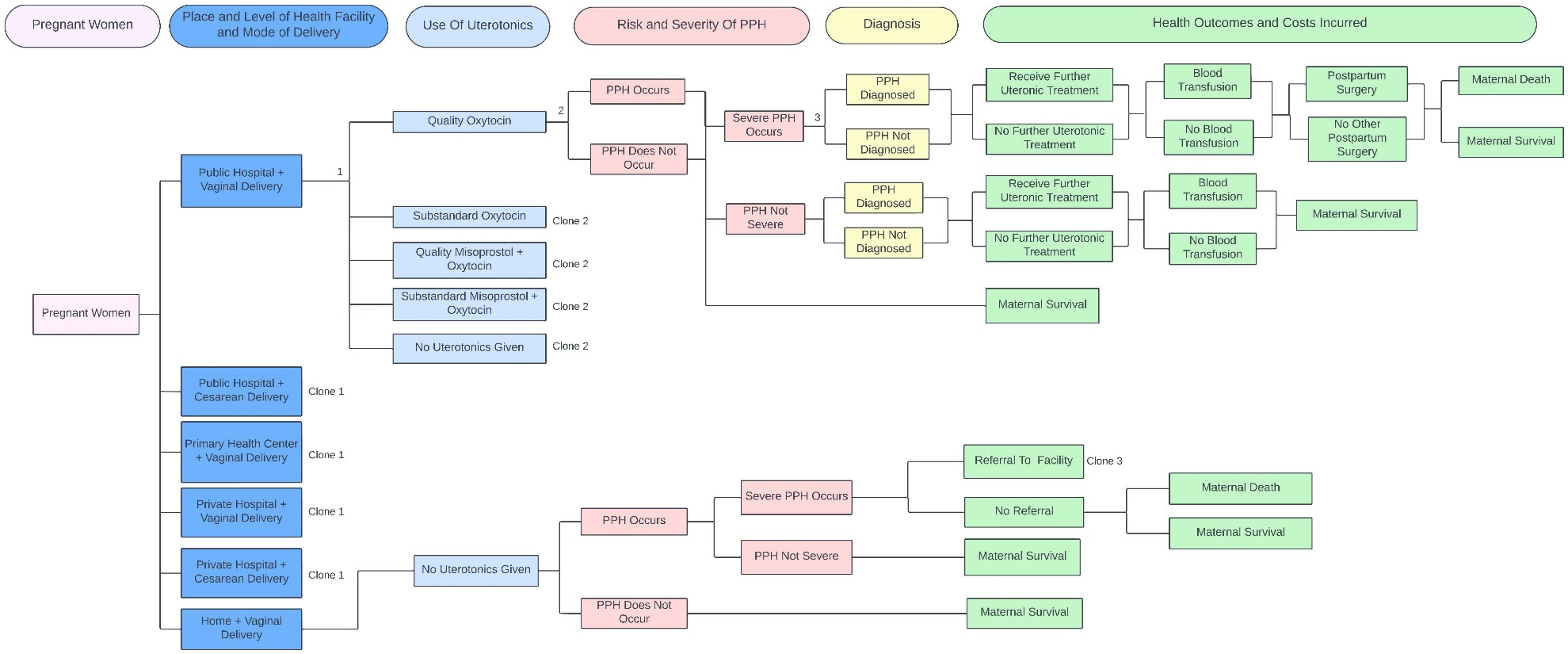

Supplement: S1 Fig — (DOCX) [file pgph.0003181.s001.docx]
