# Supplementary material for: Modeling the economic burden of postpartum hemorrhage due to substandard uterotonics in Ghana
Source: PLOS Glob Public Health. 2024 Jun 20;4(6):e0003181. doi: 10.1371/journal.pgph.0003181 (PMC11189185; doi:10.1371/journal.pgph.0003181)
Supplement: S2 Fig — Probabilistic Sensitivity Analysis was conducted for the 1,000 simulations to show annual cost savings versus total PPH case reduction by improving quality of uterotonics. The orange data point represents the baseline estimate while the blue data points are the remaining simulated estimates. (DOCX) [file pgph.0003181.s002.docx]

S2 Fig. Probabilistic Sensitivity Analysis


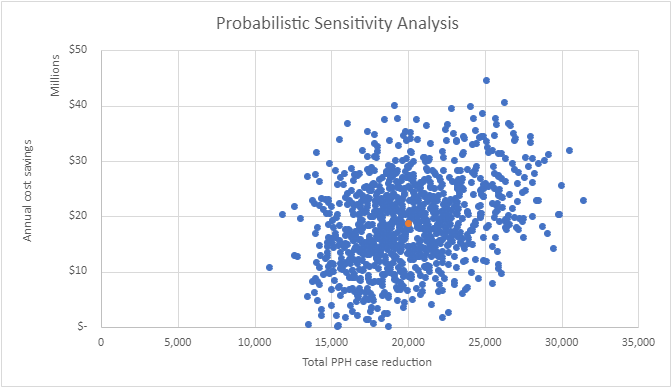


Probabilistic Sensitivity Analysis was conducted for the 1,000 simulations to show annual cost savings versus total PPH case reduction by improving quality of uterotonics. The orange data point represents the baseline estimate while the blue data points are the remaining simulated estimates.
