# Supplementary material for: Modeling the economic burden of postpartum hemorrhage due to substandard uterotonics in Ghana
Source: PLOS Glob Public Health. 2024 Jun 20;4(6):e0003181. doi: 10.1371/journal.pgph.0003181 (PMC11189185; doi:10.1371/journal.pgph.0003181)
Supplement: S2 Table — C-section = Caesarean section; DHS = Demographic and Health Survey; KOL = Key Opinion Leader; PPH = postpartum hemorrhage; USD = United States dollars; WHO = World Health Organization. (DOCX) [file pgph.0003181.s004.docx]

S2 Table. Suggested model input data to estimate burden of substandard uterotonics.

| Parameter variable | Unit | Suggested Source |
| --- | --- | --- |
| Demographic Inputs |  |  |
| Total population |  | United Nations |
| Birth rate | Per 1,000 people | United Nations |
| Maternal mortality rate | Per 100,000 live births | WHO |
| Female life expectancy at birth | Years | United Nations |
| GDP per Capita | USD | World Bank |
| Population Characteristics |  |  |
| Distribution by characteristics (if available, e.g. region/ rurality/ wealth quintile/ insurance coverage status/ age) | % | DHS |
| Care-seeking Behaviors |  |  |
| Proportion of delivery locations and delivery methods by characteristics (if available, e.g. region/ rurality/ wealth quintile/ insurance coverage status/ age) | | |
| Public Hospital and Vaginal birth | % | DHS |
| Public Hospital and C-section | % |  |
| Primary Health Center and Vaginal birth | % |  |
| Private Hospital and Vaginal birth | % |  |
| Private Hospital and C-section | % |  |
| Home | % |  |
| Proportion of diagnosis with PPH | % |  |
| Proportion of referrals among severe PPH from home and PHC | % |  |
| Utilization of Uterotonics |  |  |
| Suggested uterotonic agents |  |  |
| Oxytocin | % | Literature or KOL opinion |
| Oxytocin and Misoprostol | % |  |
| No uterotonics given | % |  |
| Carbetocin | % |  |
| Proportion of substandard uterotonics |  |  |
| Oxytocin | % | Literature or KOL opinion |
| Misoprostol | % |  |
| Carbetocin | % |  |
| Health Outcomes |  |  |
| Risk of PPH |  |  |
| PPH ≥500ml | % | Gallos et al.[1] |
| PPH ≥1000ml | % |  |
| Risk of PPH (quality vs. substandard uterotonics) |  |  |
| PPH ≥500ml | Risk ratio | E-MOTIVE trial |
| PPH ≥1000ml | Risk ratio |  |
| Proportion of receiving additional uterotonics treatment among diagnosed PPH and undiagnosed PPH |  |  |
| Quality uterotonics | % | Assumption based on E-MOTIVE trial |
| Substandard uterotonics | % |  |
| Proportion of blood transfusion among diagnosed and undiagnosed PPH |  |  |
| Quality uterotonics | % | Assumption based on E-MOTIVE trial |
| Substandard uterotonics | % |  |
| Proportion of postpartum surgery among vaginal births | % | KOL Opinion |
| Costs Inputs |  |  |
| Out-of-pocket expenses to women and their families |  |  |
| No PPH | USD | KOL Opinion |
| Mild PPH | USD |  |
| Severe PPH without surgery | USD |  |
| Severe PPH with surgery | USD |  |
| Costs to payor (if available, e.g. National Health Insurance Scheme) |  |  |
| No PPH | USD | National Health Insurance Authority |
| Mild PPH | USD |  |
| Severe PPH without surgery | USD |  |
| Severe PPH with surgery | USD |  |

C-section = Caesarean section; DHS = Demographic and Health Survey; KOL = Key Opinion Leader; PPH= postpartum hemorrhage; USD = United States dollars; WHO = World Health Organization.

Reference:

1. Gallos ID, Williams HM, Price MJ, Merriel A, Gee H, Lissauer D, et al. Uterotonic agents for preventing postpartum haemorrhage: a network meta-analysis. Cochrane Pregnancy and Childbirth Group, editor. Cochrane Database Syst Rev. 2018 [cited 8 Jun 2023]. doi:10.1002/14651858.CD011689.pub2
